# Variability of Reaction Time as a Marker of Executive Function Impairments in Fibromyalgia

**DOI:** 10.1155/2022/1821684

**Published:** 2022-07-05

**Authors:** Stefan Duschek, Cristina Muñoz Ladrón de Guevara, María José Fernández Serrano, Casandra I. Montoro, Santiago Pelegrina López, Gustavo A. Reyes del Paso

**Affiliations:** ^1^UMIT TIROL, University for Health Sciences, Medical Informatics and Technology, Institute of Psychology, Hall in Tirol 6060, Austria; ^2^Department of Psychology, University of Jaén, Jaén 23400, Spain; ^3^Department of Methodology for Behavioral Science, University of Granada, Granada 18071, Spain

## Abstract

In addition to chronic widespread pain and depression and anxiety symptoms, patients with fibromyalgia frequently experience cognitive problems. This study investigated executive functions in fibromyalgia via a Go/No-Go task. To obtain comprehensive information about performance, traditional and ex-Gaussian parameters of reaction time (RT) variability were used, in addition to speed and accuracy indices. Ex-Gaussian parameters show an excellent fit to empirical RT distributions. Fifty-two female fibromyalgia patients and twenty-eight healthy controls participated. The task included 60 visual stimuli, which participants had to respond to (Go stimuli) or withhold the response to (No-Go stimuli). After 30 trials, the task rule changed, such that previous No-Go stimuli had to be responded to. Performance was indexed by the hit rate, false alarm rate, and mean (*M*) and intraindividual standard deviation (SD) of RT and the ex-Gaussian parameters mu, sigma, and tau. Mu and sigma indicate the *M* and SD of the Gaussian distribution; tau reflects the *M* and SD of the exponential function. Patients exhibited a lower hit rate, higher *M* RT, and higher tau than controls. Moreover, patients showed greater decrease of the hit rate after the change of task rule. In the entire sample, SD, sigma, and tau were inversely associated with the hit rate and positively associated with the false alarm rate. While the greater decline in hit rate after the change in task rule indicates deficient cognitive flexibility, the lack of any difference in false alarm rate suggests intact response inhibition. Higher *M* RT reflects reduced cognitive or motor speed. Increased tau in fibromyalgia indicates greater fluctuations in executive control and more frequent temporary lapses of attention. For the first time, this study demonstrated that indices of RT variability, in particular those derived from the ex-Gaussian function, may complement speed and accuracy parameters in the assessment of executive function impairments in fibromyalgia. Optimized assessment may facilitate the personalization of therapies aimed at improving the cognitive function of those with the disorder.

## 1. Introduction

Fibromyalgia syndrome (FMS) is a chronic condition of widespread pain accompanied by symptoms like fatigue, sleep disturbance, depression, and anxiety [1, 2]. The prevalence of FMS is estimated at 2 to 4% in the general population, where women are more frequently affected than men [2]. FMS symptoms cause severe reductions in well-being and quality of life [3, 4]. Although the precise etiology of FMS remains unknown, sensitization of central nociceptive pathways and deficient pain-inhibiting mechanisms are believed to play a key role [5]. In addition to physical and emotional symptoms, FMS is frequently associated with cognitive disruption, reflected in problems with attention and memory or reduced processing speed, for example [6–8]. According to patients' reports, these difficulties can significantly affect social and professional functioning and are among the most serious symptoms of the disorder [9, 10].

The present study is concerned with executive functions in FMS. The term executive functions refer to complex cognitive abilities that enable the regulation, coordination, and sequencing of basic mental operations [11, 12]. Executive functions are essential for the control of most behaviors, and deficits therein may greatly impede activities of daily life. Several studies have documented executive function impairments in FMS. For example, patients performed worse than healthy controls on tasks assessing cognitive flexibility [13], working memory updating [6], decision-making [13, 14], mental planning [15], and arithmetic processing [16, 17]. However, negative findings have also been reported, including for response inhibition tests [18, 19] and by a study quantifying multiple executive functions [20]. Discrepancies between studies may be explained by differences in the tasks used, as well as in sample size and composition (see [15] for an overview of the findings of previous studies and a discussion of their differences).

In the present study, executive functions were assessed in FMS patients and healthy controls using a Go/No-Go task; in addition to task accuracy, intraindividual variability of reaction time (RT) was taken as an indicator of executive functions [21]. Short-term trial-by-trial RT fluctuations during cognitive tasks have been related to the coordination of cognitive operations and integrity of brain regions involved in executive functions [22, 23]. High RT variability is associated with poor performance on executive function tasks, indexed by traditional parameters like the correct response rate or RT [24–26]. Moreover, it is widely acknowledged that RT variability reflects fluctuations in executive functions; therefore, it can serve as an index of lapses in attentional control [27, 28].

The RT distribution can be described in various ways, such as by traditional and ex-Gaussian models, which provide different parameters [29–31]. Mean (*M*) and standard deviation (SD) are the most frequently used traditional indices of the RT distribution. The ex-Gaussian function is a convolution of a Gaussian (normal) and exponential function characterized by the following three parameters. Mu provides an estimate of the *M* of the Gaussian distribution; sigma is an index of the SD of the Gaussian function; tau reflects the combined *M* and SD of the exponential function, serving as an indicator of extreme values, i.e., the “right tail” of a positively skewed distribution. The ex-Gaussian distribution provides an excellent fit to empirical RT distributions [32]. In cognitive tasks, tau of RT represents unusually slow responses, which follow an exponential distribution and are closely related to short-term lapses of attention [33–35]. In contrast, mu and sigma reflect the *M* and variability of RT, irrespective of extremely slow responses [36, 37].

Intraindividual variability in RT may constitute useful information for investigations of executive function deficits in FMS; subtle impairments, which are not reflected in traditional measures such as the rate of correct responses or M RT, may be reflected in the SD, sigma, or tau of RT. Therefore, in this study, RT variability was compared between FMS patients and healthy controls. Moreover, the relationship between variability indices and task accuracy was investigated. As interindividual differences in RT variability may depend on the RT magnitude, the M RT was controlled for in the analyses (c.f. [38]).

In the Go/No-Go task, the participant is required to respond to a defined stimulus or set of stimuli (Go stimuli) and to withhold the response to another stimulus or set of stimuli (No-Go stimuli) [39]. In addition to selective attention, the task enables quantification of response inhibition [40]. As Go trials are typically more frequent than No-Go trials, the participant develops a tendency to respond, which must be suppressed during No-Go trials. Therefore, poor inhibition performance is reflected in an increased response rate to No-Go stimuli (false alarms). Moreover, the Go/No-Go task may be designed such that the task rule changes during execution, for example, by reversal of the assignment of stimuli to the Go and No-Go conditions [41]. This enables assessment of the ability to quickly adjust behavior to the new rule, i.e., cognitive flexibility.

The following main hypotheses were tested in this study: (1) lower executive function performance was expected in FMS patients than controls, reflected in a lower rate of hits (i.e., responses to Go trials), higher rate of false alarms (inhibition deficit), and greater decline in performance after the change of task rule (reduced cognitive flexibility). (2) Moreover, a longer RT was expected in FMS patients than controls, reflecting reduced speed of cognitive processing. (3) Poor executive function would also be reflected in greater intraindividual RT variability in patients than controls, i.e., higher SD for the traditional model and higher sigma and tau for the ex-Gaussian model. (4) RT variability was hypothesized to predict traditional performance parameters. Accordingly, SD, sigma, and tau were expected to be inversely associated with hit rate and positively associated with the false alarm rate. These associations should persist after controlling for RT magnitude (M or mu). In addition, to test for possible effects of comorbid depression and anxiety disorders on executive functions, task performance was compared between FMS patients suffering and not suffering from these disorders.

## 2. Materials and Methods

### 2.1. Participants

This study was part of a larger project investigating cognition and emotional processing in FMS [15, 42]. While the same sample was investigated in [15, 42], none of the data have previously been published, except for the Beck Depression Inventory (BDI) and McGill Pain Questionnaire (MPQ) scores. In total, 52 female FMS patients and 28 healthy women participated in the study. Patients were recruited via the Fibromyalgia Association of Jaén and Úbeda (Spain). All diagnoses were made by a rheumatologist according to the 2010 American College of Rheumatology (ACR) criteria for FMS [2]. Controls were recruited from voluntary and neighborhood associations. The exclusion criteria for both study groups were metabolic abnormalities, neurological disorders (e.g., traumatic head injury), and other severe somatic (e.g., cancer) or psychiatric (e.g., drug dependency and psychosis) diseases. The control group was additionally required to be free from acute or chronic pain of any kind.  Table 1 includes the demographic and clinical data of the sample.

### 2.2. Clinical Assessments

The Structured Clinical Interview for Axis I Disorders of the Diagnostic and Statistical Manual for Mental Disorders (DSM-4) (SCID, [43]) was applied to diagnose mental disorders. The severity of depressive symptoms was assessed using the Spanish version of the BDI [44] (score range: 0-63). The sum score (range: 0-146) and current pain intensity score (range: 0-5) of the MPQ (Spanish adaption [45]) were used for quantification of clinical pain. The questions of the BDI refer to the past week, including the present day; those of the MPQ refer to the present moment.

### 2.3. Cognitive Assessment

The Go/No-Go task was presented using the E-Prime software (Psychology Software Tools, Inc., Sharpsburg, PA). The task consisted of six blocks of 10 trials each. During blocks 1-3, the participants were required to press a key as quickly as possible when a Go stimulus (a letter, randomly chosen for each participant; stimulus height = 1.4 cm) appeared on the screen and to withhold the key press when a No-Go stimulus (a different letter, randomly chosen for each participant) appeared. During blocks 4-6, the instruction was reversed such that the participants had to respond to stimuli that were No-Go stimuli during blocks 1-3 and to withhold responses to previous Go stimuli. After each trial, participants received acoustic feedback on whether the response was correct or not (two tones differing in pitch and sound). Each stimulus was presented for 750 ms; the intertrial intervals were 3,000 ms. The ratio between Go trials and No-Go trials was 7/3 in all blocks. Responses were classified as hits (key press in Go trials during stimulus presentation or the following intertrial interval), false alarms (key press in No-Go trials during stimulus presentation or the following intertrial interval), missing responses (no key press in Go trials), and correct rejections (no key press in No-Go trials). Participants were instructed regarding how to perform the task, orally and in writing. Prior to the task, they were informed that the rule would change after block 3; the change was indicated by a buzzing sound during the actual task. The main parameters of task performance were the hit rate, false alarm rate, and *M* RT in each of the six blocks. In addition, intraindividual SD, mu, sigma, and tau were computed across all trials. Ex-Gaussian parameters (mu, sigma, and tau) were computed in R using the package retimes (version 3.6.2; Massida, 2013; R Core Team, 2019). Only the RTs of correct responses (hits) were included in the analysis. Anticipatory responses (RT < 200 ms) were discarded.

### 2.4. Procedure

The study was conducted over two sessions performed on two consecutive days. During the first session, a clinical psychologist recorded sociodemographic data and medication use, checked for violations of the exclusion criteria, carried out the SCID interview, and administered the self-report questionnaires. During the second session, the Go/No-Go task was performed as described previously. Participants were asked not to consume analgesic drugs, alcohol, or caffeine and not to engage in rigorous physical exercise, for 24 hours before the study. Written informed consent was obtained from all participants. The research adhered to all relevant regulations and institutional policies and was performed in accordance with the Helsinki Declaration and approved by the Ethics Committee of the University of Jaén (Spain).

### 2.5. Statistical Analysis

Statistical analyses were performed using SPSS (ver. 21.0; IBM Corp., Armonk, NY). Demographic and clinical data were compared between FMS patients and controls using *t* tests and chi-squared tests (see Table 1). For the comparison between FMS patients and controls in the performance parameters of the Go/No-Go task, ANOVAs were applied. The dependent variables were the hit rate and false alarm rate, as well as the *M*, intraindividual SD, mu, sigma, and tau of RT, averaged across all blocks of the task. In the ANOVAs for SD, sigma, and tau, *M* (for SD) and mu (for sigma and tau) values were included as covariates. Moreover, to analyze the effect of the change in task rule between blocks 3 and 4, repeated measures ANOVAs were computed for the hit rate, false alarm rate, and *M* RT, with the between-subject factor of group (FMS patients vs. controls) and within-subject factor of block (block 3 vs. block 4). Finally, univariate ANOVAs were computed to compare FMS patients suffering and not suffering from depression and anxiety disorders. Dependent variables correspond to those of the comparison between FMS patients and controls.

Linear associations between parameters were quantified, as a first step, by regression analysis including traditional performance indices (hit rate, false alarm rate, *M*, of RT) as the dependent variables and M, SD, mu, sigma, and tau as predictors, in separate regression models. Moreover, stepwise regression analyses were performed to estimate the relative contributions of traditional and ex-Gaussian parameters of RT to the variance in the hit and false alarm rates. Separate models were computed, including traditional parameters (*M* and SD of RT) and ex-Gaussian parameters (mu, sigma, and tau of RT) as predictors.

To account for possible differences in relationships between FMS patients and controls, group was used as a dummy variable in all regression models. Alpha was set at .05 in all analyses. Given the possibility of type I error inflation due to multiple statistical testing, the use of a significance threshold of 5% can be considered. However, this would substantially reduce the power of the tests, i.e., increase the chance of type II errors and reduce the probability of detecting any effects present.

## 3. Results and Discussion

### 3.1. Group Differences in Task Parameters

**Figure 1:**
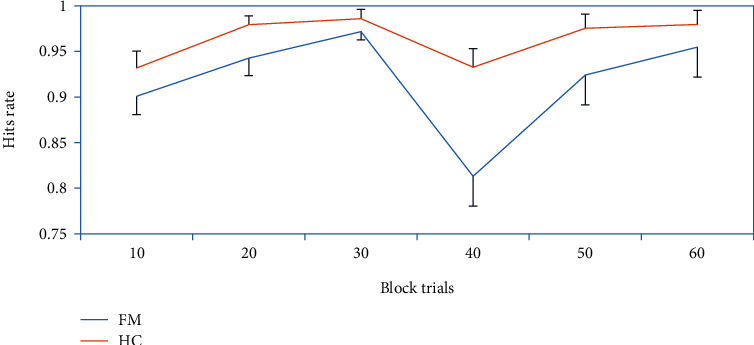
Hit rate across the six blocks of the Go/No-Go task.

FMS patients exhibited a lower overall hit rate on the task than controls, as well as a longer *M* RT and higher tau (Table 2). Group differences in the false alarm rate, mu, and sigma did not reach significance. The group difference in the intraindividual SD of RT was significant without controlling for the M RT (*p* = .017), but not after including the *M* RT as a covariate.  Figure 1 displays the hit rate across the six task blocks. While FMS patients showed a marked decrease in hit rate from blocks 3 to 4, only a slight decline was seen in controls. The ANOVA revealed a main effect of block (block 3 vs. block 4) (*F*[1, 78] = 20.57, *p* < .001, and *n*_*p*_^2^ = .21) and a group x block interaction (*F*[1, 78] = 5.09, *p* = .027, and *n*_*p*_^2^ = .06). Post hoc analysis indicated that the reduction in hit rate was significant in both groups, but with a larger effect size in FMS patients (*F* [1, 51] = 25.14, *p* < .001, and *n*_*p*_^2^ = .33) than controls (*F* [1, 27] = 4.83, *p* = .037, and *n*_*p*_^2^ = .15). The main effect of block and interaction effect were not significant for the false alarm rate or RT. The ANOVAs did not reveal differences between patients suffering and not suffering from depression or anxiety disorders (all *Fs* < .92, all *ps* > .34).

### 3.2. Linear Associations between Task Parameters

Table 3 includes the associations of hit rate, false alarm rate, and the *M* RT with the remaining task parameters in the entire sample, after controlling for the effect of group. Hit rate was inversely associated with all RT parameters except tau; the false alarm rate was positively associated with all RT parameters. Moreover, the *M* RT was positively related with all of the remaining RT parameters.

Results of the stepwise regression analyses with hit rate and false alarm rate as dependent variables and traditional RT parameters and ex-Gaussian parameters as predictors, controlling for group, are presented in Table 4. In the model for hit rate and traditional RT parameters, *M* was included in the first step and intraindividual SD in the second step. *M* was the only significant predictor of false alarm rate. Concerning ex-Gaussian parameters, mu was included in the first step of the model for hit rate; in the second step, tau further improved the predictive power. In the model for false alarm rate, tau was included as a predictor in the first step; in the second step, mu further improved the predictive power.

## 4. Discussion

This study investigated executive functions in FMS based on a Go/No-Go task. Performance was indexed by traditional parameters of task accuracy and RT and by indices of RT variability. FMS patients exhibited a lower overall hit rate and longer RT on the task than healthy controls. Moreover, they showed a greater reduction of hit rate after the change of task rule. The false alarm rate did not differ between the groups. Intraindividual RT variability, indexed by the ex-Gaussian parameter tau, was higher in patients than controls. In contrast, no group difference arose for the SD or sigma of RT. Regression analysis of the entire sample suggested that SD, sigma, and tau were inversely related to the hit rate and positively related to the false alarm rate.

### 4.1. Executive Function Impartments in Fibromyalgia Assessed via Traditional Performance Parameters

The lower hit rate in FMS patients confirms previous observations of executive function impairments in the disorder (for an overview, see [15]). While the overall hit rate constitutes a relatively nonspecific parameter of executive functions, the decline of hit rate seen after the change in task rule reflects deficits in cognitive flexibility in FMS. Between blocks 3 and 4, the hit rate decreased by approximately 15% in patients and 5% in controls, indicating that patients had greater difficulty in adjusting their behavior to the new rule. This is consistent with previous observations of reduced performance in FMS patients on the Wisconsin Card Sorting Test, which measures cognitive flexibility in terms of the ability to rapidly detect and adjust to, changing rules in a categorization task [13]. Moreover, FMS patients showed a smaller shifting index in the Five Digits Test, reflecting problems in quickly shifting between different task modes ([15]; however, see [46] for negative findings pertaining to impaired flexibility in FMS). The lack of any group difference in false alarm rate in this study suggests intact response inhibition in FMS. Previous studies on this component of executive function in FMS revealed inconsistent results. While lower performance on the Stroop test in patients pointed towards reduced inhibition capacity ([15, 46]; however, see [19] for a divergent finding), patients did not differ from controls in false alarms on a Go/No-Go task [18], nor in performance on the Multisource Interference Test [19]. Therefore, further research is warranted to achieve clarity regarding the inhibition impairments in FMS. The *M* RT was markedly longer in our FMS patients than controls, which replicates various studies demonstrating reduced cognitive speed in FMS (e.g., [16, 17, 19, 47, 48]).

### 4.2. Executive Function Impartments in Fibromyalgia Assessed via Analysis of Intraindividual RT Variability

As an alternative methodological approach, executive functions were also quantified using intraindividual trial-to-trial RT variability. To avoid confounding between RT magnitude and RT variability, RT magnitude (*M* or mu) was controlled for in the group comparison of variability parameters (c.f. [38]). While RT variability (indexed by the ex-Gaussian parameter tau) was higher in FMS patients than controls, the group difference did not reach significance for SD or sigma. It may be that tau is more sensitive to group differences than SD and sigma; moreover, the different variability parameters may relate to different aspects of cognitive performance [30]. Importantly, tau represents the *M* and variability of the exponential component of the ex-Gaussian function and thus the skew of the distribution. Therefore, tau reflects extremes in the RT distribution related to infrequent, slow responses [34, 35]. High values of tau are commonly interpreted as a manifestation of increased lapses of attentional control [49, 50]. Lapses of attention result from temporary failure in executive functions and constitute a transdiagnostic symptom of mental and physical conditions including psychotic disorders [51], attention-deficit/hyperactivity disorder [34], drug abuse [52], traumatic head injury [53], sleep disorders [54, 55], and age-related cognitive decline [25].

### 4.3. RT Variability, Brain Activity, and Psychological Factors

Intraindividual RT variability during cognitive tasks has been related to prefrontal cortex function [38]. Consistent with this hypothesis, prefrontal lesions in dementia were accompanied by increased RT variability [56]. Moreover, patients with prefrontal lesions due to traumatic head injury exhibited higher RT variability than healthy individuals and patients with nonfrontal cortical lesions [57]. The role of the prefrontal cortex in RT variability is also supported by fMRI studies [21, 38]. For example, higher RT variability during Go/No-Go tasks was associated with lower activity in prefrontal areas and the anterior cingulate [22, 58]. Event-related fMRI during a selective attention task revealed that lapses of attention are preceded by reduced activity in the right prefrontal cortex and anterior cingulate [28]. It is widely acknowledged that prefrontal activity plays a key role in executive functions [12, 59], such that RT variability may be viewed as a correlate of the neural processes underlying these abilities.

Various physiological factors have been considered to mediate increased RT variability in clinical conditions, including changes in grey matter density, white matter integrity, and catecholaminergic and cholinergic neurotransmission (for a review, see [21]). Functional interference between the neural pathways mediating pain and cognition may play a role in the increased RT variability and executive function impairments seen in FMS [15]. A central nervous system pain matrix has been identified, which includes the somatosensory cortex, anterior cingulate, insula, thalamus, and prefrontal cortex [60]. There is strong evidence implicating exaggerated activity in this network in the hyperalgesia that characterizes FMS [5]. Various neuroimaging studies demonstrated that this hyperactivity is also present in prefrontal areas [5, 61, 62]. As delineated above, activity in the prefrontal cortex is the main physiological correlate of executive functions and RT variability. Increased demands on prefrontal areas due to exaggerated nociceptive processing may reduce the neural resources available for cognition, thus leading to the observed deficits. Regarding cerebral metabolism, catecholaminergic neurotransmission may play a role. Dopamine is involved in pain inhibition, and reduced dopaminergic activity has been documented in FMS [63, 64]. In turn, dopamine is an essential transmitter for executive function processing in the prefrontal cortex [65], and deficient dopaminergic metabolism has been related to increased RT variability [21, 23].

### 4.4. Combination of Traditional Performance Indices and Ex-Gaussian Parameters of RT Variability

The suitability of intraindividual RT variability for indexing executive functions was confirmed by the relationships of variability parameters with the rate and false alarm rates seen in our entire sample. This corroborates previous reports of close associations between RT variability and performance accuracy on executive functions tasks [24–26]. According to individual regression analyses, hit rate was associated with the *M*, SD, mu, and sigma of RT, while the false alarm rate was associated with the *M*, SD, mu, tau, and sigma. When RT magnitude (*M* or mu) was controlled for in stepwise regression analysis of variability parameters, the hit rate was still significantly related to SD and tau, and the false alarm rate was still related to tau. Regarding the regression analysis of ex-Gaussian parameters, it is important to note that inclusion of mu and tau in the second step of the models led to a greater proportion of the variance in performance being explained compared to the inclusion of one of the parameters in the first step (mu for hit rate and tau for false alarm rate). This suggests that the combination of both variables may facilitate the prediction of performance. In contrast, sigma did not explain any additional variance in the hit or false alarm rate, relative to that explained by mu and tau, which suggests that this parameter may play a subordinate role in the prediction of performance.

### 4.5. Limitations

A limitation of this study was the lack of control for possible effects of medication on task performance in FMS. Such effects could be investigated by comparing subgroups distinguished according to the use of particular medications or combinations thereof; due to the small sizes of the subgroups, this was not feasible in the present study. However, most available studies comparing FMS patients using antidepressants, anxiolytics, and opiates and nonopioid analgesics with those not using these medications did not reveal differences in performance on cognitive tasks [13, 15–17, 48]. Pain medications were discontinued prior to the testing session, which may have influenced cognitive performance (via a transient increase in pain severity). The comparison between FMS patients suffering and not suffering from depression or anxiety disorders did not reveal significant differences in task performance. Although executive function impairments have been observed in patients with major depression [66], our finding is in line with previous studies suggesting that comorbid depression and anxiety disorders play a subordinate role in the cognitive impairments seen in FMS [6, 13, 17, 48, 67].

## 5. Conclusions

In conclusion, this study confirmed the presence of executive function impairments in FMS, which may be reflected in cognitive flexibility rather than response inhibition [15, 19]. Moreover, the results replicate previous observations of reduced processing speed in FMS patients (e.g., [16, 19]). The ex-Gaussian parameter tau suggested increased RT variability in FMS, reflecting fluctuations in the control of basic mental operations and temporary lapses of attention [38]. Indices of RT variability, in particular those derived from the ex-Gaussian function, may be a useful compliment to traditional parameters of speed and accuracy for investigations of executive function impairments in clinical disorders, such as fibromyalgia. The findings suggest that the combination of traditional performance indices and ex-Gaussian parameters of RT may facilitate the assessment of executive function performance. Improved assessment may in turn be useful with respect to the personalization of therapies aiming at reducing cognitive impairments, which are among the most serious symptoms of the disorder. Recent studies underline the importance of going beyond conventional medical measures (i.e., pharmacological approaches targeting symptoms) to optimize the treatment of fibromyalgia [68].

## Figures and Tables

**Table 1:** Demographic and clinical data of the sample; statistics of the group comparison.

	FMS patients (*N* = 52)	Control group (*N* = 28)	*t*[78]/*χ*^2^	*p*
Age in years (*M* ± SD)	51.25 ± 8.67	52.25 ± 6.65	.-53	.60
Years of education (*M* ± SD)	9.27 ± 3.52	10.57 ± 3.54	-1.57	.12
Body mass index (*M* ± SD)	28.29 ± 4.49	26.41 ± 4.61	1.77	.080
Depression (*N*, %)	22 (42.3)	2 (7.1)	10.72	.001
Anxiety disorder (*N*, %)	25 (48.1)	5 (17.9)	7.09	.008
Antidepressant medication (*N*, %)	27 (51.9)	2 (7.1)	15.79	*<*.001
Opioid medication (*N*, %)	23 (44.2)	0 (0.0)	17.38	*<*.001
Non-opioid analgesic medication (*N*, %)	45 (86.5)	6 (21.4)	33.39	*<*.001
Anxiolytic medication (*N*, %)	35 (67.3)	7 (25)	13.06	*<*.001
Beck Depression Inventory (*M* ± SD)	21.90 ± 12.56	4.57 ± 5.89	8.39	*<.*001
McGill Pain Questionnaire: sum score (*M* ± SD)	52.12 ± 30.31	19.50 ± 5.50	7.35	*<*.001
McGill Pain Questionnaire: pain intensity (*M* ± SD)	3.31 ± .88	1.44 ± .51	8.52	*<*.001

*Notes*. *M*: mean; SD: standard deviation; *N*: number of cases; *t*[78]: statistic of the *t* test for the group comparison (78 degrees of freedom); *χ*^2^: statistic of the chi-squared test for the group comparison; *p*: *p* value of the group comparison. Patients were using the following analgesic drugs: nonsteroidal anti-inflammatory drugs, 29 patients; paracetamol, 34 patients; metamizole, 7 patients; anticonvulsants, 10 patients; tramadol, 20 patients; and codeine, 4 patients. Thirty-six (69.2%) patients and twenty (62.5%) controls reported to be in the menopausal or premenopausal phase. Among the participants of reproductive age, the distribution of the menstrual phase was as follows: menstruation, 4 patients and 2 controls; follicular phase, 3 patients and 4 controls; ovulation phase, 3 patients and 1 control; and lutein phase, 6 patients and 5 controls.

**Table 2:** Descriptive statistics (*M* ± SD) for the task parameters; statistics of the comparison between FMS patients and controls (main effect of group in the ANOVAs). The *M* RT was controlled for in the group comparison of intraindividual SD, and mu was controlled for in the group comparisons of sigma and tau.

	FMS patients(*N* = 52)	Controls(*N* = 28)	*F*	*p*	*η*_*p*_^2^
Hit rate	.92 ± .09	.96 ± .04	6.60	.012	.08
False alarm rate	.29 ± .19	.22 ± .22	2.11	.151	.03
*M* of RT	494.50 ± 145.96	427.34 ± 78.87	5.10	.027	.06
Intraindividual SD of RT	218.99 ± 115.58	157.02 ± 91.91	1.00	.32	.01
mu of RT	293.98 ± 132.57	269.47 ± 80.02	0.80	.38	.01
sigma of RT	113.93 ± 92.45	83.15 ± 63.06	1.85	.18	.02
tau of RT	200.52 ± 102.64	157.87 ± 108.58	4.68	.034	.06

*Notes*. *M*: mean; SD: standard deviation; RT: reaction time; *N*: number of cases; *F*: statistic of the group effect in the ANOVA; *p*: *p* value of the group effect; *η*_*p*_^2^: effect size of the group effect (partial eta squared).

**Table 3:** Standardized *β* coefficients from the regression analysis conducted in the entire sample to identify predictors of the hit rate, false alarm rate, and *M* RT after controlling for the effects of group.

	Hit rate	False alarm rate	*M* of RT
*M* of RT	-.51^∗∗^	.48^∗∗^	-
Intraindividual SD of RT	-.31^∗^	.46^∗∗^	.83^∗∗^
mu of RT	-.43^∗∗^	.24^∗^	.62^∗∗^
tau of RT	-.13	.30^∗∗^	.50^∗∗^
sigma of RT	-27^∗^	.25^∗^	.72^∗∗^

*Note*. *M*: mean; SD: standard deviation; RT: reaction time. ^∗^*p* < .05; ^∗∗^*p* < .01.

**Table 4:** Statistics of the stepwise regression analyses conducted in the entire sample for predicting the hit and false alarm rates using traditional parameters (*M* and SD of RT) and ex-Gaussian parameters (mu, sigma, and tau of RT) after controlling for the effects of group.

Models for traditional parameters (*M* and SD of RT)						
Dependent variable		Predictor	*β*	*R* ^2^	*F*	*p*
Hit rate	Step 1	*M*	-.52	.33	28.53	<.001
	Step 2	*M*	-.82	.37	5.14	.026
		SD	.38			
False alarm rate	Step1	M	.48	.24	22.14	<.001

Models for ex-Gaussian parameters (mu, sigma, and tau of RT)						
Dependent variable		Predictor	*β*	*R* ^2^	*F*	*p*
Hit rate	Step 1	mu	-.43	.26	19.30	<.001
	Step 2	mu	-.54	.35	10.03	.002
		tau	-.32			
False alarm rate	Step1	tau	.30	.11	7.45	.008
	Step 2	tau	.44	.25	13.77	<.001
		mu	.40			

*Notes*. *M*: mean; SD: intraindividual standard deviation; RT: reaction time; *β*: standardized *β* coefficient for the predictor; *R*^2^: determination coefficient for the step; *F*: *F* statistic for the step; *p*: *p* value for the step.

## Data Availability

The dataset has been posted in a permanent public repository with open access and can be downloaded from this URL: https://osf.io/d9u3f/?view_only=9a05b0fa35f64f6cb32825a44f1c805d.
